# Introducing the Dapivirine Vaginal Ring in Sub-Saharan Africa: What Can We Learn from Oral PrEP?

**DOI:** 10.1007/s11904-021-00577-8

**Published:** 2021-12-15

**Authors:** Neeraja Bhavaraju, Kathleen Shears, Katie Schwartz, Saiqa Mullick, Patriciah Jeckonia, Joseph Murungu, Udita Persaud, Ashley Vij, Kristine Torjesen

**Affiliations:** 1 Afton Bloom, 123 Meserole Avenue, Brooklyn, NY 11222 USA; 2 FHI 360, 1825 Connecticut Avenue NW, Washington, DC 20009 USA; 3 FHI 360, 359 Blackwell Street, Durham, NC 27701 USA; 4 Wits Reproductive Health and HIV Institute, 8 Blackwood Avenue, Parktown, Johannesburg, South Africa; 5 LVCT Health, Batian Lane, Off Argwings Kodhek Road, Hurlingam, Nairobi, Kenya; 6 Pangaea Zimbabwe AIDS Trust, 27 Rowland Square, Milton Park, Harare, Zimbabwe; 7 United States Agency for International Development, 1300 Pennsylvania Avenue NW, Washington, DC 20523 USA

**Keywords:** HIV prevention, Pre-exposure prophylaxis, PrEP, Dapivirine vaginal ring, AGYW, Sub-Saharan Africa

## Abstract

**Purpose of review:**

Clinical trials have found that the dapivirine vaginal ring (DVR) is safe to use and effective at reducing women’s risk of acquiring HIV infection. As countries prepare for the introduction of this novel long-acting, woman-controlled prevention method, an examination of key learnings from oral pre-exposure prophylaxis (PrEP) delivery will help programs leverage successful innovations and approaches to support DVR scale-up and expand the method mix for HIV prevention.

**Recent findings:**

Intensive efforts over the past 5 years have yielded lessons on how to facilitate access to oral PrEP; expand service delivery for PrEP; address the knowledge, attitudes, and skills providers need to support PrEP initiation and effective use; develop messaging that builds community and partner support and combats stigma; and understand the cyclical nature of PrEP use.

**Summary:**

Evidence from oral PrEP introduction and scale-up can help inform and expedite DVR introduction.

## Introduction

In 2015, the Joint United Nations Programme on HIV/AIDS (UNAIDS) set targets to reduce new HIV infections to 500,000 and provide 3 million people at substantial risk of HIV with oral pre-exposure prophylaxis (PrEP) as part of combination prevention by 2020 [1, 2]. Yet in 2019, estimated new HIV infections worldwide totaled 1.7 million — over three times the 2020 UNAIDS target [3]. Global use of oral PrEP has grown significantly since it became available in 2012 but has fallen far short of the UNAIDS goal, with less than 1 million cumulative initiations by December 2020 [4].

The failure to meet these ambitious targets highlights the need for more resources, innovative approaches, and an expanded method mix to increase coverage and achieve impact with HIV prevention. This is particularly true for women, especially adolescent girls and young women (AGYW) ages 15–24 years, who bear a disproportionate HIV burden and have few HIV prevention methods they can control without engaging partners [5]. In sub-Saharan Africa (SSA), women and girls account for 59% of new HIV infections, and 83% of new HIV cases among those ages 15–19 are among adolescent girls [6]. While AGYW are extremely vulnerable to HIV transmission, they have also had challenges initiating and continuing oral PrEP use due to a myriad of factors, including stigma, negative reactions from partners or family members, daily pill burden, side effects, and inconvenience [7].

Multiple new biomedical HIV prevention products are in the development pipeline. Currently, the dapivirine vaginal ring (DVR) is ready for market introduction, with global regulatory and World Health Organization (WHO) approvals [8, 9]. The DVR is a silicone ring containing dapivirine, a non-nucleoside reverse transcriptase inhibitor (NNRTI), which is delivered intravaginally for 28 consecutive days when used as directed, works at the site of potential infection, and has low systemic absorption. Designed to address the needs of women and AGYW, the DVR can be self-inserted and used discreetly. It is also a long-acting method, with each ring worn continuously for 1 month before replacement. In clinical trials and open-label extension (OLE) studies, the DVR was found to reduce HIV-1 acquisition by 27–62% with no safety concerns or increased risk of HIV drug resistance [10–13]. Post-hoc exploratory analyses indicated up to 75–91% HIV-1 risk reduction among women whose post-use rings showed the highest levels of dapivirine drug release, suggesting that higher adherence may result in greater protection [14].

Expanding the HIV prevention method mix and increasing choice will likely increase coverage across all prevention methods and reduce HIV acquisition, based on learnings from contraception [15]. Evidence from HIV prevention clinical trials and discrete choice experiments, as well as contraceptive studies, demonstrates that women select different products based on how they “fit” within their lives, affect relationships, and provide “peace of mind” [16, 17]. With the DVR under review by multiple national regulatory agencies in SSA, policymakers, donors, and implementers are now considering the role of the DVR in HIV prevention. As they do, there are opportunities to leverage and build upon the oral PrEP experience.

Following its inclusion in WHO guidelines for all populations at substantial risk of HIV in 2015, oral PrEP was widely introduced across SSA [18]. Its delivery was often layered onto national antiretroviral therapy (ART) models, using ART supply chains, delivery channels, and monitoring systems. This approach likely accelerated the introduction of oral PrEP but may have also hindered scale-up. While providing oral PrEP alongside HIV treatment services was an effective way to serve serodifferent couples, reaching others at substantial risk — especially AGYW — proved more challenging [5, 19•]. In addition, using an ART monitoring approach based on an expectation of continuous adherence and retention propagated misunderstandings about the cyclical nature of HIV risk and oral PrEP use. Risk-based and key population-focused demand creation efforts also contributed to stigmatization and impeded efforts to normalize oral PrEP as a wellness choice for all [20].

In many countries, efforts are ongoing to redesign oral PrEP delivery to reduce stigma, improve access, and support effective use. The purpose of this review is to identify key learnings, adaptations, and innovations from oral PrEP rollout to provide a foundation for the successful introduction and scale-up of the DVR and future HIV prevention products. Five key lessons are discussed below and summarized in Table 1.

**Table 1:** Key lessons from oral PrEP that can inform DVR introduction

Lessons from oral PrEP	Application to DVR introduction	Additional DVR considerations
**Broad eligibility criteria enable PrEP access** Scaling back risk assessments and eligibility requirements to offer oral PrEP to all who requested it removed unnecessary barriers to its use and enabled programs to deliver PrEP in more accessible health facilities and community-based sites	The DVR’s safety profile offers the opportunity to make it available to all HIV-negative clients who request it from the outset, with limited requirements for laboratory testing or application of screening criteria	If found safe and feasible, use of HIV self-testing to monitor the HIV status of DVR users could further expand access to the DVR in diverse settings
**Diversified service delivery channels and approaches expand PrEP reach** The use of diverse service delivery models for oral PrEP increased access and uptake, especially among adolescent girls and young women (AGYW)	The clients most likely to benefit from the DVR are also more likely to be reached through a wide range of service delivery channels, such as family planning, antenatal and postnatal care, and youth-friendly services, including mobile or community-based services. Multi-month dispensing, task shifting, and virtual counseling approaches could support DVR delivery across channels	As a non-systemic product, the DVR could be made available in community-based settings and pharmacies more readily than oral PrEP; however, its classification by regulatory authorities will determine where and by whom the DVR can be provided
**Competent care and youth-friendly approaches support access and effective use, especially for AGYW** Addressing healthcare worker bias and strengthening culturally competent care improved oral PrEP access, especially for AGYW	Widespread, continuous efforts to sensitize healthcare workers, address bias, and strengthen culturally competent care will be particularly important to support uptake and effective use of the DVR because it is a new form of PrEP that will be unfamiliar to most potential users	Healthcare workers will also need to be equipped to discuss the use of a vaginally inserted product, the potential benefits of a long-acting product, and the differences in efficacy and side effects to help end users make informed choices among multiple HIV prevention options
**Strategic, innovative communications create an enabling environment** Campaigns to improve partner and community acceptance were necessary to complement targeted demand creation and support oral PrEP uptake and effective use	Sustained demand creation and engagement of partners, peers, and community members will be critical for the ring as a new product form. Messages about protection, wellness, empowerment, and pleasure developed for oral PrEP will be equally applicable to the DVR	New messaging will be needed to build familiarity with a vaginal product and support informed choice across products. Campaigns to increase demand for and community acceptance of the DVR will need to reflect and address locally relevant myths and misconceptions about vaginally inserted products
**Understanding and monitoring effective use of PrEP requires a new definition and framework** Adherence support and monitoring for oral PrEP have evolved to reflect growing understanding that oral PrEP users experience varying levels of risk over time and may start or stop using oral PrEP in response to changes in their relationship status or sexual behaviors	Support for and monitoring of effective use of the DVR should incorporate an understanding of varying levels of risk and potential discontinuous patterns of use. In addition, tools that put information in the hands of users (e.g., websites, text messages, WhatsApp groups, and chat bots) will likely be effective in supporting user decision-making across HIV prevention methods	Introduction of the DVR provides an opportunity to further evolve support for and monitoring of effective use across the portfolio of biomedical HIV prevention methods

## Broad Eligibility Criteria

WHO guidelines recommend that oral PrEP should be offered to anyone at substantial risk of HIV infection, prioritizing populations with an HIV incidence of three per 100 person-years or higher in the absence of oral PrEP. Many PrEP programs translated this guidance into a risk assessment tool or checklist to identify individuals at substantial risk of HIV. These tools, however, may be screening out those who are most vulnerable, due to their lack of awareness or their discomfort with disclosing risk factors [21, 22]. Some health care workers (HCWs) also feel uncomfortable asking the sensitive questions included in risk assessments, resulting in fewer potential clients screened for or offered oral PrEP [23]. Application of stringent risk assessment tools can further stigmatize oral PrEP and the individuals accessing it [5].

With new data available that demonstrate population-level reductions in HIV incidence in the context of community-wide HIV testing and universal access to oral PrEP [24•], a critical look at risk assessment is needed to inform delivery of oral PrEP and the DVR. The field has already started to evolve, with WHO and HIV prevention experts encouraging a reframing away from risk assessment toward an assessment of interest in and readiness for oral PrEP. Increasingly, an individual’s expression of interest in oral PrEP is considered a sufficient indicator of substantial risk. Research has demonstrated other effective approaches to identifying oral PrEP clients, including use of a sexually transmitted infection (STI) diagnosis as a proxy for HIV risk or use of client self-assessments [5, 25]. In addition to their expanding use in oral PrEP delivery, these alternate approaches to identifying clients should also be used for the DVR from the outset.

In addition to recommending oral PrEP for those at substantial HIV risk, the WHO guidelines also detail clinical eligibility requirements, including HIV-negative status, absence of acute HIV infection, no exposure to HIV in the prior 72 h, and no oral PrEP contraindications. Several additional tests are recommended, including serum creatinine, hepatitis B and C, pregnancy testing, and syndromic testing for other STIs at initiation, a follow-up HIV test every quarter, and a serum creatinine test every 6 months during oral PrEP use [26]. Early oral PrEP experiences highlight that the serum creatinine test, in particular, often limited oral PrEP initiation to clinical settings with on-site laboratory testing capabilities — typically a higher-level central or district hospital. Laboratory testing requirements also often posed a cost barrier because they needed to be paid out-of-pocket even when oral PrEP commodities were made available for free via national government or donor funding.

These considerations, along with evidence supporting optional or targeted renal safety monitoring [27, 28], have led to adaptation of national guidelines as oral PrEP has been scaled, especially in environments with limited HCW capacity and laboratory testing resources. In Kenya, for example, a serum creatinine test is recommended, not required, within 6 months of initiation [29]. Many other countries use urinalysis as a proxy for creatinine testing. Another promising innovation — use of HIV self-testing (HIVST) for PrEP follow-up — proved highly acceptable among HIV serodifferent couples in Kenya who were offered self-testing kits to use between quarterly clinic visits [30] and among female sex workers who expressed interested in using PrEP in Uganda and Zambia [31]. In a pilot evaluation among AGYW PrEP clients in Kenya, unassisted facility-based HIVST was feasible, reduced visit times, and was chosen over provider-initiated testing at almost 35% of clinic visits [32]. Ongoing studies on the use of HIVST to support delivery of oral PrEP will yield valuable insights [33–36].

The introduction of the DVR offers an opportunity to continue this evolution toward greater accessibility. With a strong safety profile and low risk of drug resistance [37•], the DVR requires regular HIV testing but no additional laboratory tests at initiation and follow-up. That said, the high rates of curable STIs among women using PrEP in demonstration studies and trials support the inclusion of STI testing and treatment alongside DVR delivery [38–40]. If proven safe and feasible, use of HIVST could further expand access to the DVR in diverse settings and alleviate the burden of DVR provision on health systems. Many countries where the DVR will be available are expanding access to HIVST, presenting an opportunity for alignment. The addition of the DVR also brings informed method choice into counseling discussions, which could further support the transition from checklists to conversation-based counseling centered around interest in HIV prevention, different prevention methods, and decisions to stop or switch methods.

## Diversified Service Delivery Channels and Approaches

Across many SSA countries, provision of oral PrEP was first aligned with provision of ART. In Kenya and Zimbabwe, for example, oral PrEP was offered in HIV drop-in centers, comprehensive care centers, or the opportunistic infections departments of health clinics, which had infrastructure to manage antiretroviral medications (ARVs) and providers who were already familiar with ARVs. In some countries (e.g., South Africa and Namibia), oral PrEP could be delivered only by nurses who had undergone nurse-initiated management of antiretroviral therapy (NIMART) training. However, these approaches did not effectively reach general population groups at substantial risk of HIV, including AGYW, who are unlikely to seek out HIV-specific service delivery channels, have concerns about stigma, and prefer low-barrier models for preventive care (e.g., limited travel, short wait times) [19•, 24•, 41].

In recent years, efforts have intensified to establish more diverse service delivery channels for oral PrEP. High AGYW initiation rates have been achieved when oral PrEP is offered in youth-friendly settings or via mobile or community-based services [19•, 42]. There is also growing emphasis on integration of oral PrEP with FP, STI, antenatal care (ANC), and postnatal care (PNC) services, which reach many women and AGYW at substantial risk of HIV with less associated stigma compared with ART settings. Initial experiences with integrated service delivery found integration to be feasible and acceptable to women [25, 43]. As a result, national ministries of health in Kenya, Zimbabwe, and South Africa, for example, have begun to further integrate FP and HIV prevention services, spurred in part by the need to better reach and serve AGYW.

To complement these structural changes, oral PrEP programs are expanding task shifting, allowing some elements of PrEP provision (e.g., HIV testing, risk assessment) to be managed by non-clinical HCW cadres, and offering multi-month dispensing to align visits for oral PrEP refills with visits for injectable contraception and STI testing [38, 44]. In addition, youth-friendly service delivery platforms, such as youth-friendly clinics, safe spaces, mobile clinics, and digital counseling platforms, have expanded to support oral PrEP delivery [45–50]. Efforts to establish diversified channels have been accelerated most recently by the COVID-19 pandemic, which highlighted the need to move services outside of facilities. Innovations include piloting telehealth models for virtual oral PrEP counseling, use of digital platforms, home delivery, and community-based pick-up points [46, 48, 51]. Delivery through pharmacies could also improve access to PrEP; a pilot study of pharmacy-based PrEP delivery with provider-assisted HIVST is underway in Kenya [52, 53].

DVR introduction will naturally layer onto expanding channels for oral PrEP. Women who may be most interested in DVR (those at substantial risk but not necessarily at the highest risk) are also less likely to seek out services in an HIV treatment setting. Meeting these women where they are in outpatient, FP, or ANC/PNC settings, for example, will be critical to facilitate access and acceptability [54]. As a vaginally inserted product that will likely require less laboratory testing for initiation and follow-up, the DVR also lends itself to provision by HCWs who focus on other sexual and reproductive health services, such as FP or birth-related care. Finally, as a non-systemic method, the DVR could be delivered by lower cadre HCWs and promoted as a self-care product, which would further facilitate community-based and pharmacy delivery and expand reach into rural areas that often lack access to oral PrEP. Many policymakers are already considering these options, learning from oral PrEP and seeking opportunities to build on the comparative advantages of the DVR in the context of informed choice across all available HIV prevention methods [55].

Whether the DVR can be offered through diverse delivery channels will depend on regulatory authorities’ classification, which will determine whether the ring can be delivered by HCWs with minimal HIV training, community health workers, or pharmacists, or self-administered without a prescription. The classification of the DVR will determine how and where it can be delivered and how accessible it will be, especially to women who cannot or do not want to use oral PrEP [55].

## Competent, Youth-Friendly Approaches

Across most delivery settings, HCWs are critical stakeholders in biomedical HIV prevention provision. As trusted sources of credible information, they are highly influential in user decisions about new HIV prevention methods [56, 57]. However, they can also act as a barrier to oral PrEP access, particularly for AGYW [19•, 58]. Studies of HCW attitudes and perceptions across countries have found that moral stances on sexual activity, especially for unmarried women, often influence clinical guidance. Many HCWs have concerns about risk compensation, despite lack of evidence from oral PrEP implementation. These attitudes drive lower willingness to prescribe oral PrEP to AGYW and create a clinical environment where AGYW may feel embarrassed, criticized, and judged [19•, 59]. The result has been that many women and AGYW who do seek services are reluctant to ask for oral PrEP or are not offered oral PrEP, while others are deterred from seeking it [58, 60, 61].

These challenges have required focused efforts to address the role of HCWs as gatekeepers. Many HCW training curricula now include sensitization about serving high-priority populations such as AGYW and female sex workers and guidance on nonjudgmental approaches to engaging with them. HCW knowledge, attitudes, and beliefs about PrEP can influence their willingness to offer PrEP [59, 62]; these biases can be addressed through values clarification exercises, in which providers are asked to recognize and confront the values underlying their interactions with potential oral PrEP users. Values clarification exercises are increasingly included in initial HCW training as well as ongoing supervision and mentorship [63, 64].

Addressing HCW bias, strengthening culturally competent care, and creating safe spaces that foster confidence, privacy, and confidentiality will be essential for successful DVR use by AGYW. Results from the DVR Phase III and OLE studies indicate adherence challenges among AGYW study participants that will need to be considered. To support uptake and effective use, HCWs will also need to address cultural dynamics around vaginally inserted products. Comfort with a vaginally inserted product is generally high (e.g., over 50%) but varies by age and geography [65, 66]. Evidence from the clinical trials and OLEs also suggests that potential DVR users will be confronted with a wide range of myths and misconceptions about vaginally inserted products, including links to witchcraft, potential severe side effects such as cancer and infertility, shame around menstruation, and concern about the cleanliness of the vagina [67–71]. HCWs will need to be equipped to discuss these concerns with potential users, as well as common side effects (e.g., vaginal discharge), sexual behavior, relationship dynamics and partner communications, and how to make an informed choice among HIV prevention options.

## Strategic Communications

Demand creation and community awareness building have proved critical to supporting oral PrEP scale-up. The oral PrEP experience demonstrates that targeted demand creation efforts for populations at substantial risk of HIV must be complemented by broader campaigns to improve community acceptance and support effective PrEP use [38, 72, 73]. Specific messages and tactics geared toward parents, male partners, and community leaders can promote awareness, provide accurate information, and help potential oral PrEP users — especially AGYW — gain support from important influencers [47, 74]. Strengthening the ability of AGYW to act as peer advocates for oral PrEP in their communities can also support uptake [75].

While initial demand creation efforts for oral PrEP were limited due to concerns about demand exceeding supply, those concerns were quickly dispelled as oral PrEP uptake lagged. In recent years, the countries with the largest oral PrEP programs, including Kenya, South Africa, and Zimbabwe, greatly expanded communications about oral PrEP across multiple channels, including mass media via television and radio and community outreach via launch events and peer ambassadors [76–80]. Comprehensive campaigns that combined mass media, social media, and community outreach contributed to improvements in PrEP uptake, including among AGYW [81, 82].

Oral PrEP has also yielded insights on communications messaging. Human-centered design research and end-user feedback revealed that risk-focused messaging is alienating to many in the target populations for oral PrEP. Instead, messaging that emphasizes protection, wellness, and pleasure is more effective [19•, 83, 84]. These messages address daily life priorities and are more likely to resonate with potential oral PrEP users than risk messaging [85]. Such messages also highlight themes of empowerment and self-efficacy, which are more appealing to potential PrEP users than concepts of vulnerability [5, 19•, 84]. This framing is evident, for example, in the PrEP4Youth public service announcements developed in South Africa that assert “*I Choose Protection, I Choose Control, I Choose My Future. No Apologies*” [86].

While demand creation for PrEP has improved and expanded since its initial introduction, effective approaches have been difficult to sustain due to resource constraints. These efforts will remain critical and will be especially important for the DVR, as a new product formulation. Evidence from the clinical trials and OLEs suggests that women who use the DVR find it highly acceptable, but initial reactions to the product can include confusion and expectations of discomfort [65, 74]. Potential DVR users will benefit from additional information and counseling on the DVR and engagement with peers who have used the product to support initial uptake [55, 65, 74]. In addition, early experiences with oral PrEP highlight the importance of engaging partners and community members, who are critical to establishing a support system for HIV prevention use. Although the DVR is designed to be discreet, users note partner influence to be an important driver of DVR uptake and ongoing use, including consideration of partner trust and other relationship dynamics [74, 87, 88]. As with oral PrEP, messages of health, protection, and safety resonate well with potential DVR users [55]. Messaging and information will likely need to be tailored to different cultural contexts, accounting for the prevalent myths and misconceptions about vaginally inserted products across local communities. Messaging will also need to address similarities and differences between the DVR and oral PrEP to support informed choice across the expanding method mix for HIV prevention.

## Effective Use

In the early stages of oral PrEP introduction, most programs adopted an approach to defining and monitoring follow-up based on experiences with ART that did not effectively capture the discontinuous nature of risk. Early implementation demonstrated that oral PrEP users experience “seasons of risk,” and that they choose to start or stop oral PrEP use based on changes in relationship status or sexual behavior [5, 89]. Oral PrEP users have also demonstrated that they recognize when they need protection, with evidence of users restarting oral PrEP as needed [91, 92]. Current oral PrEP indicators, however, do not accommodate changing levels of risk or the effectiveness of event-driven PrEP regimens for men who have sex with men. Use of the frameworks initially established for monitoring oral PrEP uptake and coverage continues to be challenging, making program data difficult to interpret and success hard to evaluate.

Therefore, metrics such as continuation and loss to follow-up, though relevant for ART, need to be reconceived for oral PrEP [89, 90, 93, 94]. In recent years, HIV prevention researchers have developed the concept of “prevention effective adherence,” with high adherence to oral PrEP during periods of risk as the goal that should guide oral PrEP counseling, follow-up, and monitoring [22]. The addition of another biomedical prevention method raises the opportunity to improve indicators for oral PrEP and the DVR and to establish measurement approaches to define “total coverage” across all PrEP modalities, acknowledging that users may switch between methods, as with contraception.

Interventions adapted from HIV treatment to support effective use, such as adherence clubs and text messages, have seen mixed results in terms of impact, but were highly acceptable and feasible to deliver at scale among AGYW, pregnant and postpartum women, and female sex workers [5, 45, 95, 96]. These approaches will need to be further optimized and adapted for the DVR, as adherence considerations for a long-acting method will differ from those for a daily pill. In addition, adherence support will need to consider different dynamics — for example, more intensive counseling on a new product formulation and information to support continued DVR use during menses or sexual intercourse.

## Conclusion

With sufficient investment and effort to build on the learnings from oral PrEP introduction and scale-up, the DVR has significant potential to expand the market for HIV prevention and contribute to reductions in HIV incidence, especially for women and AGYW who bear a disproportionate HIV burden in SSA [97]. Many of these lessons were anticipated even in the early stages of oral PrEP introduction, in part based on experiences with ART [98]. However, the persistence of HIV prevention challenges speaks to the immense investment of time, attention, and resources required to adapt large, diverse systems to effectively deliver a new category of biomedical HIV prevention methods. As a result, continued innovation and improvement will be needed, informed not only by the history of HIV treatment and prevention, but also by models developed for contraception and self-care that will yield additional insights to inform further progress. With multiple new HIV prevention products on the horizon, it is essential that we apply the lessons learned from oral PrEP to support expanded choice and coverage through new product introduction while continuing to scale up established methods.
